# A drug-related Good Samaritan Law and calling emergency medical services for drug overdoses in a Canadian setting

**DOI:** 10.1186/s12954-021-00537-w

**Published:** 2021-08-26

**Authors:** Soroush Moallef, JinCheol Choi, M.-J. Milloy, Kora DeBeck, Thomas Kerr, Kanna Hayashi

**Affiliations:** 1 Faculty of Health Sciences, Simon Fraser University, 400-1045 Howe Street, Vancouver, BC V6Z 2A9 Canada; 2 British Columbia Centre On Substance Use, St. Paul’s Hospital, Vancouver, BC Canada; 3 Department of Medicine, University of British Columbia, Vancouver, BC Canada; 4 School of Public Policy, Simon Fraser University, Burnaby, BC Canada

**Keywords:** 911-calling, Good Samaritan Law, Emergency medical services, Drug overdose

## Abstract

**Background:**

People who use drugs (PWUD) are known to fear calling emergency medical services (EMS) for drug overdoses. In response, drug-related Good Samaritan Laws (GSLs) have been widely adopted in the USA and Canada to encourage bystanders to call emergency medical services (EMS) in the event of a drug overdose. However, the effect of GSLs on EMS-calling behaviours has been understudied. We sought to identify factors associated with EMS-calling, including the enactment of the Canadian GSL in May 2017, among PWUD in Vancouver, Canada, a setting with an ongoing overdose crisis.

**Methods:**

Data were derived from three prospective cohort studies of PWUD in Vancouver in 2014–2018. Multivariable logistic regression was used to determine factors associated with EMS-calling among PWUD who witnessed an overdose event. An interrupted time series (ITS) analysis was employed to assess the impact of GSL on monthly prevalence of EMS-calling.

**Results:**

Among 540 eligible participants, 321 (59%) were males and 284 (53%) reported calling EMS. In multivariable analysis, ever having administered naloxone three or more times (adjusted odds ratio [AOR] 2.00; 95% confidence interval [CI] 1.08–3.74) and residence in the Downtown Eastside (DTES) neighbourhood of Vancouver (AOR 1.96; 95% CI 1.23–3.13) were positively associated with EMS-calling, while living in a single occupancy hotel (SRO) was negatively associated with EMS-calling (AOR 0.51; 95% CI 0.30–0.86). The post-GSL enactment period was not associated with EMS-calling (AOR 0.81; 95% CI 0.52–1.25). The ITS found no significant difference in the monthly prevalence of EMS-calling between pre- and post-GSL enactment periods.

**Conclusion:**

We observed EMS being called about half the time and the GSL did not appear to encourage EMS-calling. We also found that individuals living in SROs were less likely to call EMS, which raises concern given that fatal overdose cases are concentrated in SROs in our setting. The link between many naloxone administrations and EMS-calling could indicate that those with prior experience in responding to overdose events were more willing to call EMS. Increased efforts are warranted to ensure effective emergency responses for drug overdoses among PWUD.

**Supplementary Information:**

The online version contains supplementary material available at 10.1186/s12954-021-00537-w.

## Introduction

Drug overdoses are now one of the leading cause of accidental deaths in Canada and the USA [1, 2]. In most drug overdose cases, death occurs as a result of hypoxia, a condition requiring the attention of emergency medical services (EMS) as complications can easily arise [3, 4]. Of concern, people who use drugs (PWUD) are known to have fear about EMS-calling as calling an emergency number 911 in Canada and the USA can attract police to the scene [4–11]. Previous studies have reported possible repercussions associated with calling EMS among PWUD, including arrest and harassment by the police, and a subsequent loss of publicly funded housing or custody of children [7, 8, 12–14]. Thus, many PWUD report managing overdose situations independently by using first aid measures (e.g., cardiopulmonary resuscitation) and/or naloxone, a pharmacological antidote to an opioid overdose [6, 9, 12, 15]. However, multiple naloxone administrations are needed against potent opioids due to naloxone’s short duration of action [16–18]. Also, owing to the increased presence of highly potent synthetic opioids (i.e., fentanyl) in the unregulated drug supply [19–21], there exists a considerable need for EMS to attend suspected overdose cases [16, 19–23].

In response, Canada and 46 states in the USA have enacted drug-related Good Samaritan Laws (GSLs) aimed at reducing fear of legal repercussions when an overdose occurs through the provisions of some legal amnesties [24, 25]. In May 2017, the “Good Samaritan Drug Overdose Act” (GSA) was federally enacted in Canada to provide immunity from the arrest, charge, or prosecution of drugs possessed for personal use and related breach of conditions (e.g., probation orders, parole) when EMS is called to an overdose event [24, 25]. The protections of GSLs in the USA are similar, but vary by state [25]. To date, the body of evidence on the effectiveness of these laws is mixed [26–28]. Studies show that PWUD have low levels of knowledge of these laws and the effect of GSLs on EMS-calling behaviours has been understudied [26]. A study in Indiana, USA, found that awareness of the state’s GSL was associated with having called 911 among lay overdose responders (*n* = 217) [29]. However, this study did not account for potential confounders and the measurement of “awareness” (whether individuals had heard of the law) cannot discern whether participants had knowledge of the protections provided by the law. In a study in New York, USA, correct knowledge of the state’s GSL was associated with calling EMS compared to those with incorrect knowledge, after adjusting for socio-demographic factors [12]. However, this study measured knowledge among individuals immediately after receiving overdose rescue training that included education of the GSL, and not among PWUD in community settings.

Within the past two decades, research has identified several factors associated with EMS-calling among PWUD, including: being trained to administer naloxone [30], having administered naloxone [29], having previously witnessed an overdose [9], having a female bystander at the scene of the overdose [9], when the overdose victim is male [31], and when rescue breathing is performed [31]. In contrast, those with prior overdose experience and the presence of four or more bystanders were less likely to call EMS [9]. Other reasons for not calling EMS included: no ownership of a cell phone [14, 32], fear of endangering personal relations [8, 12], and if the overdose occurred in a private setting [6, 7, 12, 31].

The current study sought to compare the prevalence of calling EMS before and after the enactment of the GSA among community-recruited PWUD who witnessed an overdose event. We also sought to identify a range of individual factors (e.g., experience using naloxone, drug use patterns, accurate knowledge of the GSA) and social–structural exposures (e.g., negative encounters with police, sex work, drug dealing, incarceration) that may be associated with calling EMS. To guide this exploratory analysis, we drew on Rhodes’ Risk Environment framework [33]. This framework conceptualizes drug-related harm as a product of individuals interacting with macro- and micro-levels of various physical, social, economic, and political environmental factors [33]. In this context, an individual’s decision to call EMS when an overdose occurs is influenced by interactions with many dimensions of the risk environment. For instance, research has shown that PWUD are deterred from calling EMS when an overdose occurs (individual-level factor) as a result of police presence and aggressive law enforcement practices at overdose events (micro-social environment) [8, 11, 13], which is driven by the structural criminalization of PWUD (macro-policy environments) [33]. We further explored the relationship between accurate knowledge of the GSA and calling EMS during the post-enactment period. Our study constitutes an important exploratory step needed to understand the social and structural exposures that may shape EMS-calling behaviour among community-recruited PWUD in Vancouver, an epicentre of the ongoing drug poisoning crisis in Canada.

## Methods

### Study setting and design

Data were drawn from three ongoing prospective cohort studies of PWUD in Vancouver, Canada: the Vancouver Injection Drug Users Study (VIDUS), the AIDS Care Cohort to evaluate Exposure to Survival Services (ACCESS), and the At-Risk Youth Study (ARYS). VIDUS enrols HIV-seronegative adults (≥ 18 years of age) who injected unregulated drugs in the month prior to enrolment. ACCESS enrols HIV-seropositive adults who used an unregulated drug other than or in addition to cannabis (which was a controlled substance during the study period) in the month prior to enrolment. ARYS enrols street-involved youth aged 14 to 26 years who used an unregulated drug other than or in addition to cannabis in the month prior to enrolment. The studies use harmonized data collection and follow-up procedures to allow for merged data analyses. All three cohorts are administered harmonized questionnaires by trained interviewers at equal follow-up frequency (i.e., every six months). At the end of each study visit, participants receive a $40 CAD (Canadian dollar) honorarium. Further details of the three cohorts are available elsewhere [34–36]. All three cohorts have received ethics approval from the University of British Columbia/Providence Health Care Research Ethics Board.

In the primary analysis, data collected between December 2014 to May 2016 (“pre-enactment period”) and June to November 2018 (“post-enactment period”) were utilized, with a gap in data between May 2016–May 2018 due to the witnessed overdose and response questions being removed from the questionnaire during this period. Questions used to evaluate the accuracy of knowledge of the GSA (described below) were added to the post-enactment period questionnaire.

### Primary outcome measure

The primary outcome of interest was a binary measure (yes vs. no) of “Called EMS” derived from participants who responded “yes” to the question: “Have you witnessed an overdose in the last six months?” and responded “I called 911” to the subsequent question: “What happened in response to this last time?”. Other possible responses to this question included: “I administered Narcan”, “Someone else called 911”, “Person came to on their own”, “I helped”, **“**Someone else helped”, “Ambulance came”, “I left”, or “I was at a supervised consumption site”. Participants could select as many responses as appropriate. We used descriptive statistics to summarize these other responses to the witnessed overdose event among those who called EMS.

### Study sample

**Fig. 1:**
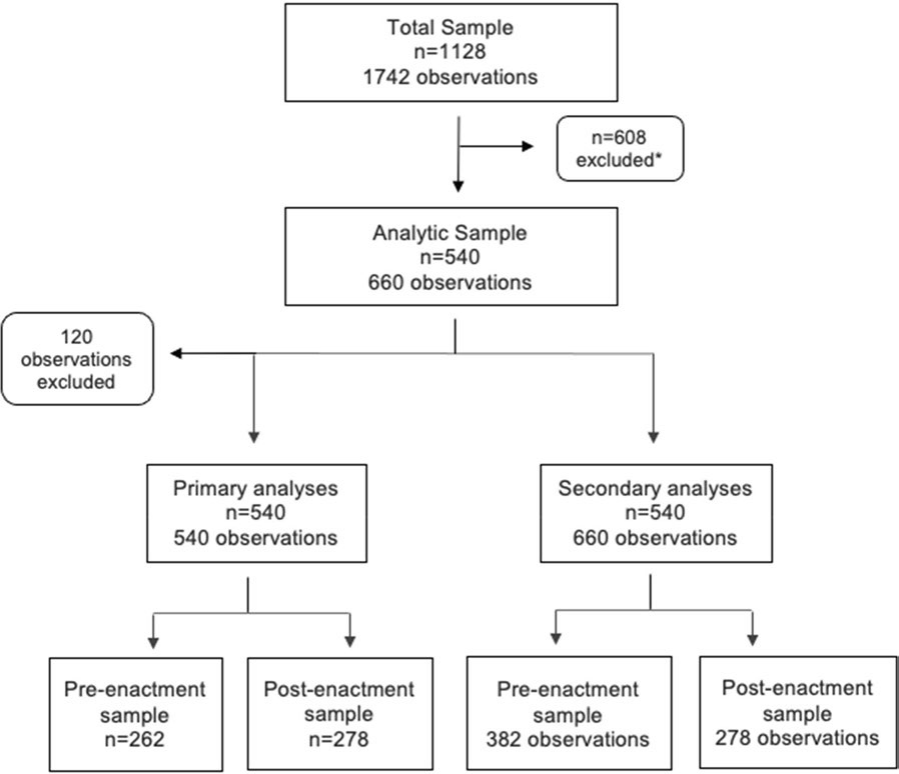
Schematic diagram of the study sample and analyses. Between December 2014 to May 2016 and June 2018 to November 2018, 1128 people who use drugs in Vancouver, Canada had witnessed an overdose event. *The sample was further restricted by the exclusion of witnessed overdose events (*n* = 608) where the respondent did not call EMS, but EMS arrived at the scene or was not needed. For the primary cross-sectional analyses, we utilized the most recent observations only among the analytic sample (120 observations excluded) to identify factors associated with EMS-calling. For the secondary analyses, which involved an interrupted time series analysis, we utilized the full 660 observations of the analytic sample and divided the observations into pre- and post-enactment of the Good Samaritan Drug Overdose Act samples

The present study included individuals who had who had witnessed an overdose event and used any drugs in the past six months during the study period. The sample was further restricted by the exclusion of witnessed overdose events where the respondent did not call EMS, but EMS arrived at the scene or was not needed (details shown in Fig. 1). Thus, the following overdose responses were excluded: “Someone else called 911”, “Ambulance came”, “At a supervised consumption site”, and “Person came to on their own”. We excluded these events as they did not require the respondent to call EMS. Based on these criteria, the starting sample of 1128 participants was restricted to 540 participants, who provided a total of 660 observations. Among the 540 participants, 101 (18.7%) provided more than one observation, with 50 (9.3%) participants contributing observations in both the pre- and post-enactment periods and 51 (9.4%) contributing repeat measurements only in the pre-enactment period. For the primary analyses (described below), because only the minority (9.3%) provided observations in both the pre- and post-enactment periods, we restricted the analytic sample to the participant’s most recent observation (120 observations excluded, and 540 observations remained) to conduct a cross-sectional analysis. These participants were divided into pre- (*n* = 262) and post-enactment (*n* = 278) samples. For the secondary analyses (described below), we utilized the full 660 observations.

### Primary analyses: factors associated with calling EMS

For this part of the analyses, the analytic sample only included the participants’ most recent observation as the majority of the sample only contributed one observation during the study period. Covariates were selected based on prior literature [9, 14, 29–32], and the hypothesized relationships between the explanatory variables and calling EMS according to the Risk Environment framework [33]. The explanatory variables of interest included the following socio-demographic characteristics: age (continuous); ancestry (white vs. non-white); gender (male vs. non-male); education (< secondary school education vs. ≥ secondary school education); residence in the Downtown Eastside neighbourhood of Vancouver (DTES), an area known as an epicentre of unregulated drug use and marginalization in British Columbia [37]; place of residence (homeless vs. single room occupancy [SRO] accommodations vs. other [e.g., apartment, house, no fixed address]). Drug use related variables included: injection drug use (≥ daily vs. < daily); use of heroin (≥ daily vs. < daily); use of stimulants (≥ daily vs. < daily), defined as powder/crack cocaine or crystal methamphetamine; use of cannabis (≥ daily vs. < daily); and ever experienced a non-fatal overdose. Other social/structural exposures included: involved in drug dealing; witnessed a known person’s overdose; ever had a negative police encounter (stopped, searched or detained by the police); ever experienced incarceration; involved in sex work (exchanged sex for gifts, food, shelter, clothes, or money); and ever administered naloxone, derived from the question: “Have you administered Narcan/naloxone to anyone in the last six months?” and the subsequent question: “(If yes) How many times?”. Responses were then coded as: “Did not administer” (reference category), “one or two times”, or “three or more times”. We also included a variable of “time” to adjust for any time-related effects between the pre- (time = 0) and post-enactment period (time = 1). All behavioural variables referred to the past six months and all variables were coded as yes versus no, unless otherwise stated. We used bivariable and multivariable logistic regression to identify factors associated with calling EMS. Time was included into the multivariable model to adjust for its effect on other covariates.

### Secondary analyses: the impact of the GSA on EMS-calling

An interrupted time series analysis with segmented regression was used to evaluate the impact of the GSA enactment on the monthly prevalence of EMS-calling (in percentage), using all eligible observations [38, 39]. After dividing the analytic sample data into pre- and post-intervention segments, linear regression was used to compute the change in intercept post-enactment to measure the prevalence level change of EMS-calling pre- and post-enactment, as well as trends in the level change (measured by changes in the slope) following the GSA enactment [38, 39]. The linear regression did not control for factors in the primary analysis. Seasonality was accounted for using the Webel–Ollech overall seasonality test, with no evidence of seasonality observed (*p* = 0.542). To account for autocorrelation, we examined the residuals and autocorrelation function plots of the prevalence of EMS-calling, with no abnormalities observed in both plots. We further conducted a Breusch–Godfrey test and observed no evidence of autocorrelation in the prevalence of EMS-calling (*p* = 0.499) [39]. We also tested for differences in sample characteristics among the pre- and post-enactment samples using Pearson’s Chi-squared test and Fisher’s test for counts < 5 (for categorical variables) or the Mann–Whitney test (for continuous variables) as appropriate.

In the sub-analysis, among the post-enactment sample, we also evaluated the accuracy of “knowledge of the GSA”, we asked participants: “Imagine you witnessed an overdose in a public place. 911 is called and the police come to the scene. Do you think the police can legally arrest you if: you have a small amount of drugs on you (scenario A), you have a larger amount of drugs on you or items (scale, etc.) that may look like you are involved in drug dealing (scenario B), and you are in a red/no-go zone you received for a previous charge that was not simple drug possession (scenario C)”. This question was created in consultation with a local lawyer who has expert knowledge about the GSA and informed by public educational material on the GSA that was created and disseminated by the local lawyer’s group [40]. Participants were categorized as having accurate knowledge of the GSA if they identified that scenario A was the only instance where the police could not legally arrest when EMS is called to an overdose (due to protections provided by the GSA) [24]. We examined the association between this variable and the outcome using the Pearson’s Chi-squared test.

All p-values were two-sided. All statistical analyses were performed using R, version 3.6.3 (R Foundation for Statistical Computing, Vienna, Austria).

## Results

### Sample characteristics

A total of 660 observations reported by 540 participants were included in the analytic sample (Fig. 1). The median number of questionnaires completed by these participants was 1 (quartile 1–3:1–1, range 1–4). Using the most recent observations, 262 (48.5%) of 540 participants reported witnessing an overdose event in the pre-enactment period and 278 (51.2%) in the post-enactment period. Among this sample (Table 1), 284 (52.6%) reported calling EMS at the most recent overdose event. The sample included 321 (59.4%) males, 288 (53.3%) of white ethnicity/ancestry, and the median age was 40.4 (quartile 1–3: 28–52) years. Other characteristics are presented in Table 1. Among the 284 participants who called EMS, 213 (75.0%) reported that the ambulance arrived at the most recent overdose scene (Table 2). In addition to calling EMS, 111 (39.1%) reported helping the individual (e.g., first aid), 102 (35.9%) reported administering naloxone, and 28 (9.9%) reported someone else helped the individual.

**Table. 1:** Characteristics of people who use drugs who witnessed an overdose in Vancouver, British Columbia, between 2014 and 2018 (*n* = 540)

Characteristic	Total (%) (*n* = 540)	Called EMS (%) (*n* = 284, 53%)	Did not call EMS (%) (*n* = 256, 47%)
Age			
Median (IQR)	40.4 (28–52)	42.6 (31–53)	35.2 (26–52)
Per year older		–	–
White (vs. non-white)	286 (53.0)	152 (53.5)	134 (52.3)
Male (vs. non-male)	321 (59.4)	167 (58.8)	154 (60.2)
< Secondary school education	287 (53.2)	167 (58.8)	140 (54.7)
DTES residency^a^	325 (60.2)	192 (67.6)	133 (52.0)
Place of residence^a^			
Homeless	130 (24.1)	66 (23.2)	64 (25.0)
Single room occupancy	252 (46.7)	129 (45.4)	123 (48.1)
Other (e.g., apartment, house, no fixed address)	158 (29.3)	89 (31.3)	69 (27.0)
Ever incarcerated	464 (85.9)	250 (88.0)	214 (83.6)
Ever had a negative police encounter^b^	361 (66.9)	250 (88.0)	171 (66.8)
Ever administered naloxone^*a*^			
Did not administer	273 (50.6)	129 (45.4)	144 (56.3)
One or two times	141 (26.1)	83 (29.2)	58 (22.7)
Three or more	81 (15.0)	50 (17.6)	31 (12.1)
Witnessed a known person overdose^ac^	310 (57.4)	169 (59.5)	141 (55.1)
Ever experienced an overdose^a^	387 (71.7)	206 (72.5)	181 (70.7)
Involved in the sex trade^ad^	71 (13.2)	40 (14.1)	31 (12.1)
Involved in drug dealing^a^	141 (26.1)	77 (27.1)	64 (25.0)
Injection drug use	243 (45.0)	136 (47.9)	107 (41.8)
Heroin^e^	207 (38.3)	113 (39.8)	94 (36.7)
Stimulants, defined as powder or crack cocaine or crystal methamphetamine^e^	200 (37.0)	110 (38.7)	90 (35.2)
Cannabis	165 (30.6)	76 (26.8)	89 (34.8)
Post-enactment period	278 (51.8)	146 (51.4)	132 (51.6)

Reference category = Did not call EMS

*EMS* Emergency medical services, *DTES* Downtown Eastside, *GSA* Good Samaritan Drug Overdose Act, *PWUD* People who use drugs

^a^Denotes behaviours and events in the past six months

^b^Police encounter refers to being stopped, searched or detained by the police

^c^A known person includes a sex partner or a friend

^d^Sex trade refers to exchanged sex for gifts, food, shelter, clothes, or money

^e^Injection or non-injection drug use

**Table. 2:** Other responses to the reported witnessed overdose event among those who called EMS (*n* = 284)

Response	*n* = 284 (%)
I called EMS and the ambulance came	213 (75.0%)
I called EMS and I helped (e.g., provided first aid)	111 (39.1%)
I called EMS and administered naloxone	102 (35.9%)
I called EMS and someone else helped	28 (9.9%)
I called EMS only	17 (6.0%)
I called EMS and I was at an OPS/SCS	13 (4.6%)
I called EMS and the person came on their own	9 (3.2%)
I called EMS and then I left	1 (0.4%)

Participants could provide more than one response

*EMS* Emergency medical services, *OPS* Overdose prevention site, *SCS* Supervised consumption site

### Primary analyses

In the multivariable logistic regression analysis (Table 3), residency in the DTES (adjusted odds ratio [AOR] 1.96; 95% confidence interval [CI] 1.23–3.13), living in a SRO (AOR 0.51; 95% CI 0.30–0.86), and ever administering naloxone three or more times (AOR 2.00; 95% CI 1.08–3.74) were significantly and positively associated with EMS-calling.

**Table. 3:** Bivariable and multivariable logistic regression analysis of factors associated with EMS-calling among PWUD in Vancouver, British Columbia (*n* = 540)

Characteristic	Unadjusted odds ratio (95% CI)	*p* value	Adjusted odds ratio (95% CI)	*p* value
*Age*				
Per year older	1.02 (1.01–1.03)	0.004	1.02 (1.00–1.03)	0.090
White (vs. non-white)	1.02 (0.73–1.44)	0.897	1.09 (0.72–1.63)	0.690
Male (vs. non-male)	0.88 (0.61–1.27)	0.493	0.91 (0.58–1.42)	0.683
< Secondary school education	0.91 (0.64–1.27)	0.570	0.85 (0.56–1.27)	0.424
DTES residency^a^	1.93 (1.36–2.74)	< 0.001	1.96 (1.23–3.13)	0.005
*Place of residence*^*a*^				
Homeless	0.80 (0.50–1.27)	0.347	0.71 (0.38–1.31)	0.278
Single room occupancy	0.81 (0.54–1.21)	0.310	0.51 (0.30–0.86)	0.013
Other (e.g., apartment, house, no fixed address)	Reference		Reference	–
Ever incarcerated	1.44 (0.89–2.36)	0.140	1.14 (0.63–2.06)	0.666
Ever had a negative police encounter^b^	1.00 (0.70–1.44)	0.979	0.93 (0.60–1.45)	0.764
*Ever administered naloxone*^*a*^				
Did not administer	Reference	–	Referenc**e**	–
One or two times	1.60 (1.06–2.42)	0.026	1.50 (0.94–2.43)	0.093
Three or more	1.80 (1.09–3.01)	0.023	2.00 (1.08–3.74)	0.029
Witnessed a known person overdose^ac^	1.20 (0.85–1.69)	0.299	1.08 (0.71–1.65)	0.714
Ever experienced an overdose^a^	1.09 (0.75–1.59)	0.637	0.85 (0.54–1.34)	0.498
Involved in the sex trade^ad^	1.18 (0.71–1.96)	0.520	1.19 (0.62–2.30)	0.605
Involved in drug dealing^a^	1.12 (0.76–1.64)	0.577	1.06 (0.64–1.76)	0.815
*At least daily drug use*				
Injection drug use	1.28 (0.91–1.80)	0.156	1.02 (0.63–1.67)	0.925
Heroin^e^	1.14 (0.80–1.61)	0.464	1.11 (0.67–1.82)	0.689
Stimulants^ef^	1.17 (0.82–1.66)	0.390	1.11 (0.71–1.75)	0.651
Cannabis	0.68 (0.47–0.98)	0.037	0.72 (0.46–1.12)	0.144
Post-enactment period	0.99 (0.71–1.39)	0.971	0.81 (0.52–1.25)	0.344

*DTES* Downtown Eastside, *EMS* Emergency medical services, *PWUD* People who use drugs

Reference category = Did not call EMS

^a^Denotes behaviours and events in the past six months

^b^Police encounter refers to being stopped, searched or detained by the police

^c^A known person includes a sex partner or a friend

^d^Sex trade refers to exchanged sex for gifts, food, shelter, clothes, or money

^e^Injection or non-injection drug use

^f^Stimulants refer to powder or crack cocaine or crystal methamphetamine

### Secondary analyses

**Fig. 2:**
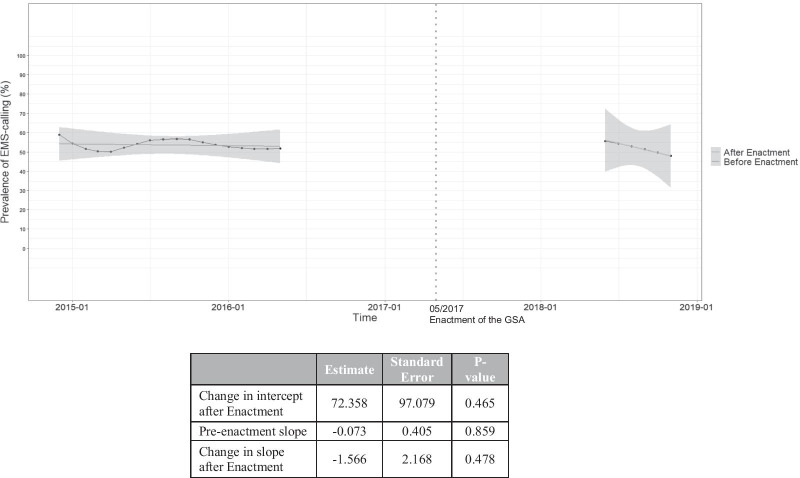
An interrupted time series analysis: calling emergency medical services (EMS) and the Good Samaritan Drug Overdose Act (GSA)

As shown in Fig. 2, the prevalence of EMS-calling in May 2016 (pre-enactment of the GSA) was 52.9% (95% CI 44.6–61.4). A year following enactment of the GSA in June 2018, the prevalence of EMS-calling was 56.2% (95% CI 42.7–69.6). In the interrupted time series analysis, we did not observe a significant level change (p = 0.465) between the pre- and post-enactment periods. There was also no statistically significant evidence in the change of the slope (*p* = 0.478) following enactment, compared to the pre-enactment slope (*p* = 0.859).

Between the pre- and post-enactment samples, we observed significant differences (all *p* < 0.05) in the following variables (pre-enactment vs. post-enactment): age (median: 38.3 vs. 42.8), < secondary school education (58.4% vs. 48.2%), place of residence (homeless: 27.5% vs. 20.9%; SRO: 48.6 vs. 45.0%; other 24.1% vs. 34.2%), ever administered naloxone (did not administer: 62.2% vs. 39.6%; one or two times: 21.8% vs. 30.2%; three or more times: 7.6% vs. 21.9%), witnessed a known person overdose (67.1% vs. 48.2%), involved in drug dealing (30.9% vs. 21.6%), and ever had a negative police encounter (74.1% vs. 60.1%) (as shown in the Web Appendix).

In the sub-analysis, 278 participants responded to the post-enactment period questionnaire (data not shown). The characteristics among this sample remained similar to the overall sample (Table 1), except a higher proportion of participants reported ever administering naloxone three or more times (82, 29.5%). Among this sample, 154 (55.4%) individuals reported calling EMS at the most recent overdose event, and 81 (29.1%) were identified as having accurate knowledge of the GSA. The prevalence of EMS-calling was 51 (33.1%) among those who had accurate knowledge of the GSA and 30 (24.2%) among those who did not with no evidence of statistically significant difference (*p* = 0.095).

## Discussion

In this study of 540 community-recruited PWUD who witnessed an overdose event, EMS was called approximately half of the time, which is similar to rates reported in other settings [11]. In the multivariable analysis, those who reported residence in the DTES, living in a SRO and three or more naloxone administrations were more likely to call EMS at the witnessed overdose event. In the interrupted time series analysis, no statistically significant differences in the monthly prevalence of EMS-calling between pre- and post-GSA enactment periods were observed. In the sub-analysis, less than one third of those who witnessed an overdose had accurate knowledge of the GSA, and there was no statistically significant relationship between accurate knowledge of the GSA and EMS-calling.

We found that those who resided in the DTES were more likely to call EMS, which is a finding contrary to past research in this setting. Specifically, a recent ethnographic study found that in response to regular police presence in the DTES, some PWUD felt compelled to choose between responding to the overdose independently, not responding or risking potential arrest by recruiting EMS [41]. In this regard, the DTES may not be an environment conducive to EMS-calling for PWUD. However, our finding may reflect another characteristic of this neighbourhood, which is the concentration of many low-threshold harm reduction and health services sites where many PWUD work as peer workers in this neighbourhood [42, 43]. Specifically, DTES residents may be more likely to work as peer workers and be aware of the importance of calling EMS or used to calling EMS through their work.

We also found that overdose witnesses who reported living in SROs were less likely to call EMS compared to those living in other private residences (e.g., apartment, house, no fixed address). Although we cannot determine the setting of the witnessed overdose event in our study, the British Columbia Coroners Service report that private residences and SROs have been the most common places where fatal overdoses occurred [44]. Indeed, studies in Canada and the USA have shown that EMS is less likely to be called when an overdose occurs in a private setting [6, 7, 12, 44, 45]. Our finding suggests that there may be characteristics of SROs that additionally hinder EMS-calling compared to other private residences. In particular, SRO tenants are subject to restrictions that are not typical to other privately housed people, including limits to guests, curfews, and codes of conduct (e.g., “crime free addendums”) [46]. In this regard, qualitative research has shown that the living environment of SROs do not meet the survival needs of PWUD in our setting [46]. In addition, this qualitative study documented many reports of unlawful evictions that forced people into homelessness [46]. This fear of losing housing and homelessness has also been documented to be a reason for PWUD in Denver, Colorado to avoid EMS-calling [8]. A recent study in our setting also found that only a quarter of those living in SROs had accurate knowledge of the GSA and a considerable portion of the sample had overestimated the protections provided by the GSA [28]. Taken together with our findings, the living environment and building policies of SROs seem to undermine the aims of the GSA. To support EMS-calling in SROs there is a need to reduce the fear of eviction through policy reform that aims to increase tenancy security and align housing policies with the aim of the GSA [46]. Another possible intervention could be a tenant- or peer-led overdose response team [47], where individuals are trained in overdose response and provided access to naloxone. A pilot project in our setting has demonstrated the feasibility and acceptability of this programme among PWUD living in SROs [47].

In addition, we found that those who had ever administered naloxone three or more times were more likely to report calling EMS. Among those who called EMS, over a third (35.9%) reported also administering naloxone at the witnessed overdose event. Our finding could be conceived as an ineffective overdose response that necessitated calling EMS [48, 49]. This interpretation is consistent with another study in British Columbia, Canada, that found that bystanders who administered three or more naloxone ampoules at an overdose event were more likely to call EMS [31]. In addition, PWUD in New York, USA, have reported delaying engaging EMS as they thought they could handle the situation independently [6]. However, our finding could also be indicative of the amount of experience PWUD have in managing an overdose situation. For instance, those who administer naloxone frequently could be more comfortable or better recognize the need to call EMS, but research is needed to confirm this.

To our knowledge, this is the first study to longitudinally evaluate temporal changes in the monthly prevalence of calling EMS during an overdose following the enactment of the GSA. Our findings show no statistically significant changes in the monthly prevalence of EMS-calling between the pre- and post-GSA enactment periods. Further, in the post-enactment period, we observed no statistically significant link between having accurate knowledge of the GSA and calling EMS. In addition, low levels of accurate knowledge of the GSA were found among those who witnessed an overdose in the sub-analysis, warranting additional education of this law. Given the mixed findings with regard to the effectiveness of GSLs [26], our findings underscore the need for future research to investigate the effectiveness of these laws. Our findings also suggest that additional measures are needed to strengthen GSL policies to increase the appeal to PWUD. One possible method to strengthen these laws is to include legal immunities for drug trafficking charges. Over a quarter of our sample (26.1%) had reported being involved in drug dealing, which may indicate an increased reluctance to call EMS given the lack of immunities provided for drug trafficking charges. Given that many PWUD are engaged in drug dealing for survival [50, 51], legal immunities for drug dealing are warranted. Taken together, innovative strategies are needed to reduce all possible repercussions associated with EMS-calling for PWUD, as these have been shown to cause PWUD to delay and avoid EMS-calling [6–8, 12–14].

During our study period, there was a significant increase in the number of overdose-related deaths and a policy change by the Vancouver Police Department (VPD) that could have influenced the findings. Specifically, the British Columbia Coroners Service reported an approximately fourfold increase in overdose-related deaths per year (369 to 1550) between 2014 and 2018 [52]. As per VPD’s non-attendance policy at overdose events [53], the VPD states that attendance at overdose events will only occur if the overdose results in a fatality or if EMS has requested for their assistance. Thus, it is conceivable that police presence at overdose events increased over our study period with the increase in the number of fatal overdoses, but further research is needed to confirm this. In addition, in January 2018, the VPD increased routine police surveillance in the DTES, where the majority of our participants reported residence in [54]. This action has been shown to undermine the aims of the GSA and exacerbate PWUD’s mistrust with EMS-calling [41]. In this way, it is unclear whether the increased police surveillance affected EMS-calling rates and undermined the impact of the GSA in our setting. Further attention is needed to align police practices with the aim of the GSA.

Our findings have several limitations. Our measurement of knowledge was not validated among participants. Nonetheless, we note that it was created in consultation with a local lawyer with expert knowledge about the GSA and informed by public educational material on the GSA that was created and disseminated by the local lawyer’s group for public legal education purposes [40]. In the interrupted time series analysis, the gap in our data corresponds to a full year before and after the enactment of the GSA. This reduces our ability to make inferences of the longitudinal impact of the GSA on EMS-calling. However, the post-enactment period data used are consistent with another longitudinal study that had no gap in data and lagged GSA policy in the year the law was enacted to account for potential lagged effects of implementation [55]. Secondly, we also observed significant differences in some sample characteristics between the pre- and post-enactment samples that may have introduced some bias into our study (see Additional file 1). Although we are unable to determine the direction of bias, the pre-enactment sample was younger and exhibited more markers of social marginalization (i.e., higher levels of homelessness, living in SROs, negative encounters with the police, and involvement in drug dealing) and had less experience administering naloxone than the post-enactment sample. Considering our findings that those who lived in SROs were less likely to call EMS, and those who had experience administering naloxone many times were more likely to call EMS, we hypothesize that the post-enactment sample would have been more likely to call EMS compared to the pre-enactment sample. However, we did not find a significant association between the post-enactment period and EMS-calling even in bivariable analyses. This may reflect some threats to history that were not accounted for, such as the increased police surveillance in the DTES during the post-enactment period (discussed previously). Thirdly, due to the cross-sectional study design used in the multivariable analysis, the temporal relationship between the exposures and the outcome are indeterminate. In addition, the selective nature of our sample reduces the ability for our results to be generalized to all PWUD. Further, the majority of our measures were self-reported, which may introduce some response bias into our study. Specifically, we are unable to determine the direction of bias for those self-reporting EMS-calling, although the rates reported in our study are similar to those found among PWUD in other settings [11]. In addition, self-reported measures have been shown to be generally reliable and valid among PWUD [56–58].

## Conclusion

In this study, we assessed factors associated with EMS-calling and the impact of a GSL among PWUD in a setting with a community-wide overdose crisis. Among our sample, EMS was called about half the time, with those who administered naloxone three or more times more likely to call EMS. Of concern, individuals living in SROs were less likely to call EMS, indicating the need to support EMS-calling among SRO residents. This may include policy reform to increase tenancy security during medical emergencies and considerations of a tenant- or peer-led overdose response team at SROs. We did not observe statistically significant differences in the rates of EMS-calling pre- and post-enactment of the GSA or in the prevalence of accurate knowledge of the GSA between those who did and did not call EMS. Further research is needed to establish the effectiveness of these laws and to examine EMS-calling among PWUD in other settings. As the overdose crisis continues, further effort is also urgently needed to increase EMS-calling rates, including reducing all possible repercussions associated with EMS-calling.

## Supplementary Information

**Additional file 1**. Characteristics of people who use drugs who witnessed an overdose stratified by pre- and post-enactment of the Good Samaritan Drug Overdose Act.

## Data Availability

The data used for this study are not publicly available and can be requested from the corresponding author on reasonable request and with permission of the University of British Columbia/Providence Health Care Research Ethics Board.

## References

[1] Centers for Disease Control and Prevention. Increases in Drug and Opioid-Involved Overdose Deaths - United States, 2010–2015. https://www.cdc.gov/mmwr/volumes/65/wr/mm655051e1.htm. Published 2016. Accessed July 25, 2018.

[2] Statistics Canada. Causes of death, 2017. https://www150.statcan.gc.ca/n1/daily-quotidien/190530/dq190530c-eng.htm. Published 2017. Accessed February 25, 2020.

[3] Sporer KA (1999). Acute heroin overdose. Ann Intern Med.

[4] Warner-Smith M, Darke S, Lynskey M, Hall W (2001). Heroin overdose: Causes and consequences. Addiction.

[5] Seal KH, Kral AH, Gee L (2001). Predictors and prevention of nonfatal overdose among street-recruited injection heroin users in the San Francisco Bay Area, 1998–1999. Am J Public Health.

[6] Tracy M, Piper TM, Ompad D (2005). Circumstances of witnessed drug overdose in New York City: Implications for intervention. Drug Alcohol Depend.

[7] Klassen D, Buxton J. Overdose Recognition and Response in the BC Take Home Naloxone Program. Review of data up to July 2016. http://www.bccdc.ca/resource-gallery/Documents/EducationalMaterials/Epid/Other/THNreportAug_final.pdf. Published 2016.

[8] Koester S, Mueller SR, Raville L, Langegger S, Binswanger IA (2017). Why are some people who have received overdose education and naloxone reticent to call Emergency Medical Services in the event of overdose?. Int J Drug Policy.

[9] Tobin KE, Davey MA, Latkin CA (2005). Calling emergency medical services during drug overdose: an examination of individual, social and setting correlates. Addiction.

[10] Lankenau SE, Wagner KD, Silva K (2013). Injection drug users trained by overdose prevention programs: responses to witnessed overdoses. J Community Health.

[11] Clark AK, Wilder CM, Winstanley EL (2014). A systematic review of community opioid overdose prevention and naloxone distribution programs. J Addict Med.

[12] Jakubowski A, Kunins HV, Huxley-Reicher Z, Siegler A (2018). Knowledge of the 911 Good Samaritan Law and 911-calling behavior of overdose witnesses. Subst Abus.

[13] Latimore AD, Bergstein RS (2017). “Caught with a body” yet protected by law? Calling 911 for opioid overdose in the context of the Good Samaritan Law. Int J Drug Policy.

[14] Follett K, Piscitelli A, Parkinson M, Munger F. Barriers to Calling 9-1-1 during Overdose Emergencies in a Canadian Context. *Crit Soc Work*. 2019;15(1).

[15] Ambrose G, Amlani A, Buxton JA (2016). Predictors of seeking emergency medical help during overdose events in a provincial naloxone distribution programme: A retrospective analysis. BMJ Open.

[16] Boyer EW (2012). Management of opioid analgesic overdose. N Engl J Med.

[17] Rzasa Lynn R, Galinkin JL (2018). Naloxone dosage for opioid reversal: current evidence and clinical implications. Ther Adv Drug Saf.

[18] Faul M, Lurie P, Kinsman JM, Dailey MW, Crabaugh C, Sasser SM (2017). Multiple naloxone administrations among emergency medical service providers is increasing. Prehosp Emerg Care.

[19] Centers for Disease Control and Prevention. Drug Overdose Deaths. https://www.cdc.gov/drugoverdose/data/statedeaths.html. Published 2018.

[20] Government of Canada. Overview of national data on opioid-related harms and deaths. https://www.canada.ca/en/health-canada/services/substance-use/problematic-prescription-drug-use/opioids/data-surveillance-research/harms-deaths.html. Published 2018.

[21] Tupper KW, McCrae K, Garber I, Lysyshyn M, Wood E (2018). Initial results of a drug checking pilot program to detect fentanyl adulteration in a Canadian setting. Drug Alcohol Depend.

[22] Lewis CR, Vo HT, Fishman M (2017). Intranasal naloxone and related strategies for opioid overdose intervention by nonmedical personnel: a review. Subst Abuse Rehabil.

[23] Canadian Centre on Substance Use and Addiction. Calling 911 in Drug Poisoning Situations. 2017.

[24] Government of Canada. About the Good Samaritan Drug Overdose Act. https://www.canada.ca/en/health-canada/services/substance-use/problematic-prescription-drug-use/opioids/about-good-samaritan-drug-overdose-act.html. Published 2017. Accessed November 1, 2018.

[25] National Conference of State Legislatures. Drug Overdose Immunity and Good Samaritan Laws. http://www.ncsl.org/research/civil-and-criminal-justice/drug-overdose-immunity-good-samaritan-laws.aspx. Published 2019.

[26] Moallef S, Hayashi K (2020). The effectiveness of drug-related Good Samaritan laws: A review of the literature. Int J Drug Policy.

[27] Schneider KE, Park JN, Allen ST, Weir BW, Sherman SG (2020). Knowledge of Good Samaritan laws and beliefs about arrests among persons who inject drugs a year after policy change in Baltimore, Maryland. Public Health Rep.

[28] Moallef S, DeBeck K, Milloy MJ, Somers J, Kerr T, Hayashi K (2021). Knowledge of a drug-related Good Samaritan law among people who use drugs, Vancouver, Canada. Health Educ Behav.

[29] Watson DP, Ray B, Robison L (2018). Lay responder naloxone access and Good Samaritan law compliance: postcard survey results from 20 Indiana counties. Harm Reduct J.

[30] Zadoretzky C, McKnight C, Bramson H (2017). The New York 911 Good Samaritan law and opioid overdose prevention among people who inject drugs. World Med Heal Policy.

[31] Karamouzian M, Kuo M, Crabtree A, Buxton JA (2019). Correlates of seeking emergency medical help in the event of an overdose in British Columbia, Canada: Findings from the Take Home Naloxone program. Int J Drug Policy.

[32] Davidson PJ, Ochoa KC, Hahn JA, Evans JL, Moss AR (2002). Witnessing heroin-related overdoses: the experiences of young injectors in San Francisco. Addict.

[33] Rhodes T (2009). Risk environments and drug harms: a social science for harm reduction approach. Int J Drug Policy.

[34] Strathdee SA, Palepu A, Cornelisse PGA (1998). Barriers to use of free antiretroviral therapy in injection drug users. J Am Med Assoc.

[35] Wood E, Kerr T, Marshall BDL (2009). Longitudinal community plasma HIV-1 RNA concentrations and incidence of HIV-1 among injecting drug users: Prospective cohort study. BMJ.

[36] Wood E, Stoltz JA, Montaner JSG, Kerr T (2006). Evaluating methamphetamine use and risks of injection initiation among street youth: the ARYS study. Harm Reduct J.

[37] Linden IA, Mar MY, Werker GR, Jang K, Krausz M (2013). Research on a vulnerable neighborhood-the Vancouver downtown eastside from 2001 to 2011. J Urban Health.

[38] Wagner A, Soumerai S, Zhang F, Ross-Degnan D (2002). Segmented regression analysis of interrupted time series studies in medication use research. J Clin Pharm Ther.

[39] Bernal JL, Cummins S, Gasparrini A (2016). Interrupted time series regression for the evaluation of public health interventions: a tutorial. Int J Epidemiol.

[40] Pivot Legal Society. The Good Samaritan drug overdose act: what you need to know. http://www.pivotlegal.org/fact_sheet_what_you_need_to_know_about_the_good_samaritan_drug_overdose_act. Published 2017. Accessed March 3, 2020.

[41] Collins AB, Boyd J, Mayer S (2019). Policing space in the overdose crisis: a rapid ethnographic study of the impact of law enforcement practices on the effectiveness of overdose prevention sites. Int J Drug Policy.

[42] Kennedy MC, Boyd J, Mayer S, Collins A, Kerr T, McNeil R (2019). Peer worker involvement in low-threshold supervised consumption facilities in the context of an overdose epidemic in Vancouver, Canada. Soc Sci Med.

[43] Neil B, Campbell L, Culbert L. *A Thousand Dreams: Vancouver’s Downtown Eastside and the Fight for Its Future*. D&M; 2010.

[44] BC Coroners Service. *Illicit Drug Overdose Deaths in BC*.; 2018. https://www2.gov.bc.ca/assets/gov/birth-adoption-death-marriage-and-divorce/deaths/coroners-service/statistical/illicitdrugoverdosedeathsinbc-findingsofcoronersinvestigations-final.pdf.

[45] Ambrose G, Amlani A, Buxton JA (2016). Predictors of seeking emergency medical help during overdose events in a provincial naloxone distribution programme: a retrospective analysis. BMJ Open.

[46] Fleming T, Damon W, Collins AB, Czechaczek S, Boyd J, McNeil R (2019). Housing in crisis: a qualitative study of the socio-legal contexts of residential evictions in Vancouver’s Downtown Eastside. Int J Drug Policy.

[47] Bardwell G, Fleming T, Collins AB, Boyd J, McNeil R (2019). Addressing intersecting housing and overdose crises in Vancouver, Canada: opportunities and challenges from a tenant-led overdose response intervention in single room occupancy hotels. J Urban Heal.

[48] Bohnert ASB, Nandi A, Tracy M (2011). Policing and risk of overdose mortality in urban neighborhoods. Drug Alcohol Depend.

[49] Darke S, Hall W (2003). Heroin overdose: research and evidence-based intervention. J Urban Heal Bull N Y Acad Med.

[50] DeBeck K, Shannon K, Wood E, Li K, Montaner J, Kerr T (2007). Income generating activities of people who inject drugs. Drug Alcohol Depend.

[51] Sherman SG, Latkin CA (2002). Drug users’ involvement in the drug economy: implications for harm reduction and HIV prevention programs. J Urban Health.

[52] BC Coroners Service. Illicit Drug Toxicity Deaths in BC. 2019:21. https://www2.gov.bc.ca/assets/gov/birth-adoption-death-marriage-and-divorce/deaths/coroners-service/statistical/illicit-drug.pdf.

[53] Vancouver Police Department Planning and Research Section. 11.04 Guidelines for Police Attending Illicit Drug Overdoses. https://vancouver.ca/police/policeboard/documents/0648DrugOverdosePolicy2006Jun14.pdf. Published 2006. Accessed March 3, 2020.

[54] Vancouver Police Department. Vancouver police work to increase safety in the Downtown Eastside. https://mediareleases.vpd.ca/2018/01/30/vancouver-police-work-to-increase-safety-in-the-downtown-eastside/. Published 2018. Accessed April 9, 2020.

[55] McClellan C, Lambdin BH, Ali MM (2018). Opioid-overdose laws association with opioid use and overdose mortality. Addict Behav.

[56] Napper LE, Fisher DG, Johnson ME, Wood MM (2010). The reliability and validity of drug users’ self reports of amphetamine use among primarily heroin and cocaine users. Addict Behav.

[57] Needle R, Fisher DG, Weatherby N (1995). Reliability of self-reported HIV risk behaviors of drug users. Psychol Addict Behav.

[58] Darke S (1998). Self-report among injecting drug users: a review. Drug Alcohol Depend.

