# Supplementary material for: A drug-related Good Samaritan Law and calling emergency medical services for drug overdoses in a Canadian setting
Source: Harm Reduct J. 2021 Aug 26;18:91. doi: 10.1186/s12954-021-00537-w (PMC8393452; doi:10.1186/s12954-021-00537-w)
Supplement: Supplementary file 1 — Additional file 1. Characteristics of people who use drugs who witnessed an overdose stratified by pre- and post-enactment of the Good Samaritan Drug Overdose Act. [file 12954_2021_537_MOESM1_ESM.docx]

| **Table 1. Characteristics of people who use illicit drugs who witnessed an overdose in Vancouver, British Columbia (n=540) stratified by pre- (December 2014 – May 2016) and post-enactment (June 2018 to November 2018) of the Good Samaritan Drug Overdose Act.** | | | | |
| --- | --- | --- | --- | --- |
| **Characteristic** | **Total (%)**  **(n=540)** | **Pre-enactment (%)**  **(n = 262, 49%)** | **Post-enactment (%)**  **(n = 278, 51%)** | **P-value** |
| Age |  |  |  |  |
| Median (IQR) | 40.4 (28-52) | 38.3 (26-51) | 42.8 (31-53) | <0.001 |
| White (vs non-white) | 286 (53.0) | 149 (56.9) | 137 (49.3) | 0.100 |
| Male (vs non-male) | 321 (59.4) | 162 (61.8) | 159 (57.2) | 0.649 |
| < Secondary school education | 287 (53.2) | 153 (58.4) | 134 (48.2) | 0.037 |
| DTES residency^a^ | 325 (60.2) | 155 (59.2) | 170 (61.2) | 0.701 |
| Place of residence^a^: |  |  |  | 0.023 |
| Homeless | 130 (24.1) | 72 (27.5) | 58 (20.9) |  |
| Single room occupancy | 252 (46.7) | 127 (48.5) | 125 (45.0) |  |
| Other (e.g., apartment, house, no fixed address) | 158 (29.3) | 63 (24.1) | 95 (34.2) |  |
| Ever incarcerated | 464 (85.9) | 217 (82.8) | 247 (88.9) | 0.059 |
| Ever had a negative police encounter^b^ | 361 (66.9) | 194 (74.1) | 167 (60.1) | <0.001 |
| Ever administered naloxone^a^: |  |  |  | <0.001 |
| Did not administer | 273 (50.6) | 163 (62.2) | 110 (39.6) |  |
| One or two times | 141 (26.1) | 57 (21.8) | 84 (30.2) |  |
| Three or more | 81 (15.0) | 20 (7.6) | 61 (21.9) |  |
| Witnessed a known person overdose^ac^ | 310 (57.4) | 176 (67.1) | 134 (48.2) | <0.001 |
| Ever experienced an overdose^a^ | 387 (71.7) | 183 (69.95) | 204 (73.4) | 0.415 |
| Involved in the sex trade^ad^ | 71 (13.2) | 37 (14.1) | 34 (12.2) | 0.577 |
| Involved in drug dealing^a^ | 141 (26.1) | 81 (30.9) | 60 (21.6) | 0.018 |
| Injection drug use | 243 (45.0) | 119 (45.4) | 124 (44.6) | 0.917 |
| Heroin^e^ | 207 (38.3) | 101 (38.56) | 106 (38.1) | 0.991 |
| Stimulants, defined as powder or crack cocaine or crystal methamphetamine^e^ | 200 (37.0) | 94 (35.9) | 106 (38.1) | 0.651 |
| Cannabis | 165 (30.6) | 88 (33.6%) | 77 (27.7) | 0.220 |

IQR: Interquartile range. DTES: Downtown Eastside.

^a^ Denotes behaviours and events in the past six months

^b^ Police encounter refers to being stopped, searched or detained by the police

^c^ A known person includes a sex partner or a friend

^d^ Sex trade refers to exchanged sex for gifts, food, shelter, clothes, or money

^e^ Injection or non-injection drug use
